# Supplementary figures and images for: Identification of clinically approved small molecules that inhibit growth and affect transcript levels of developmentally regulated genes in the African trypanosome
Source: PLoS Negl Trop Dis. 2020 Mar 13;14(3):e0007790. doi: 10.1371/journal.pntd.0007790 (PMC7094864; doi:10.1371/journal.pntd.0007790)

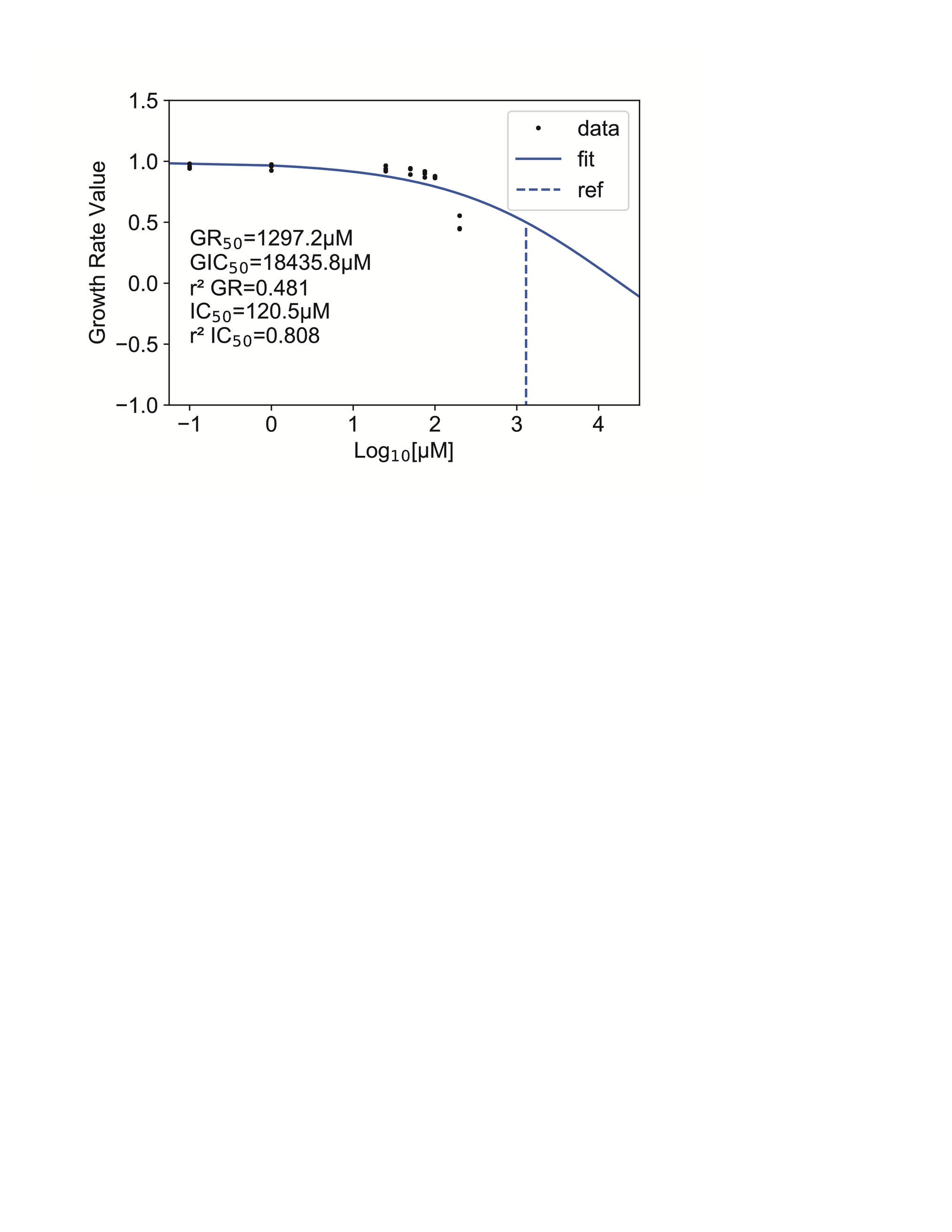

Supplement: S1 Fig — Percent growth inhibition over a range of concentrations for the indicated drug. Data were fitted and indicated values calculated using the GRMetrics R package. GR50, the concentration at which the effect reaches a growth rate (GR) value of 0.5 based on interpolation of the fitted curve (dashed lines). GIC50, the drug concentration at half-maximal effect for calculated growth rate. r2 GR, the coefficient of determination for how well the GR curve fits to the data points. IC50, the concentration at which relative cell count = 0.5. r2 IC50, the coefficient of determination for how well the traditional curve fits to the data points. (TIF) [file pntd.0007790.s001.tif]

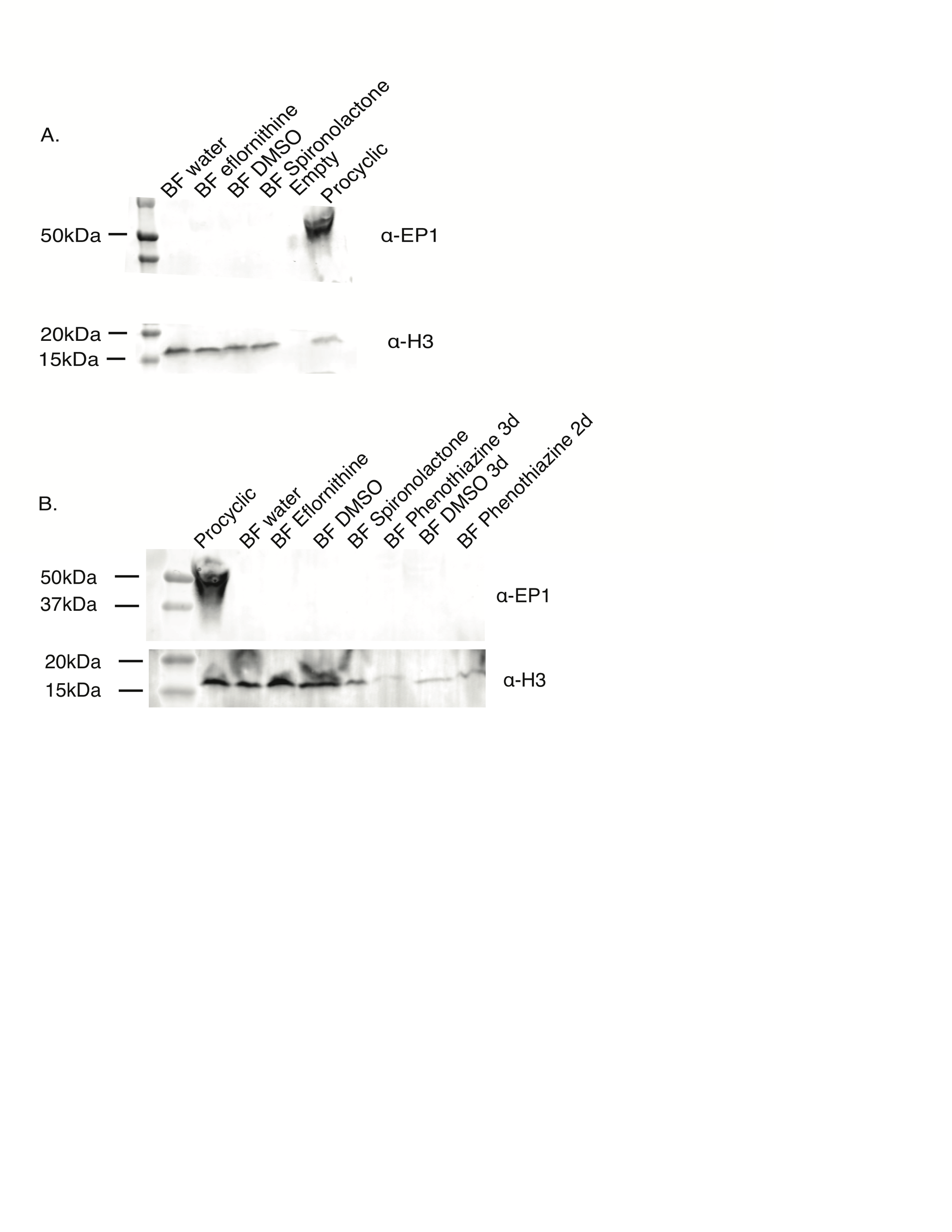

Supplement: S2 Fig — A) Anti-procyclin western blot for parasites isolated after 3d of treatment with the indicated drug. Procyclic parasites were used as a positive control. Anti-H3 was used as a loading control. Note that the samples ran high toward the right-hand side of the blot. B) Anti-procyclin western blot for parasites isolated after 2d or 3d of treatment with the indicated drug. Procyclic parasites were used as a positive control. Anti-H3 was used as a loading control. Because phenothiazine-treated parasites were so sick it was difficult to get sufficient numbers of cells for the assay; consequently, these samples and their controls are loaded with less protein. (TIF) [file pntd.0007790.s002.tif]

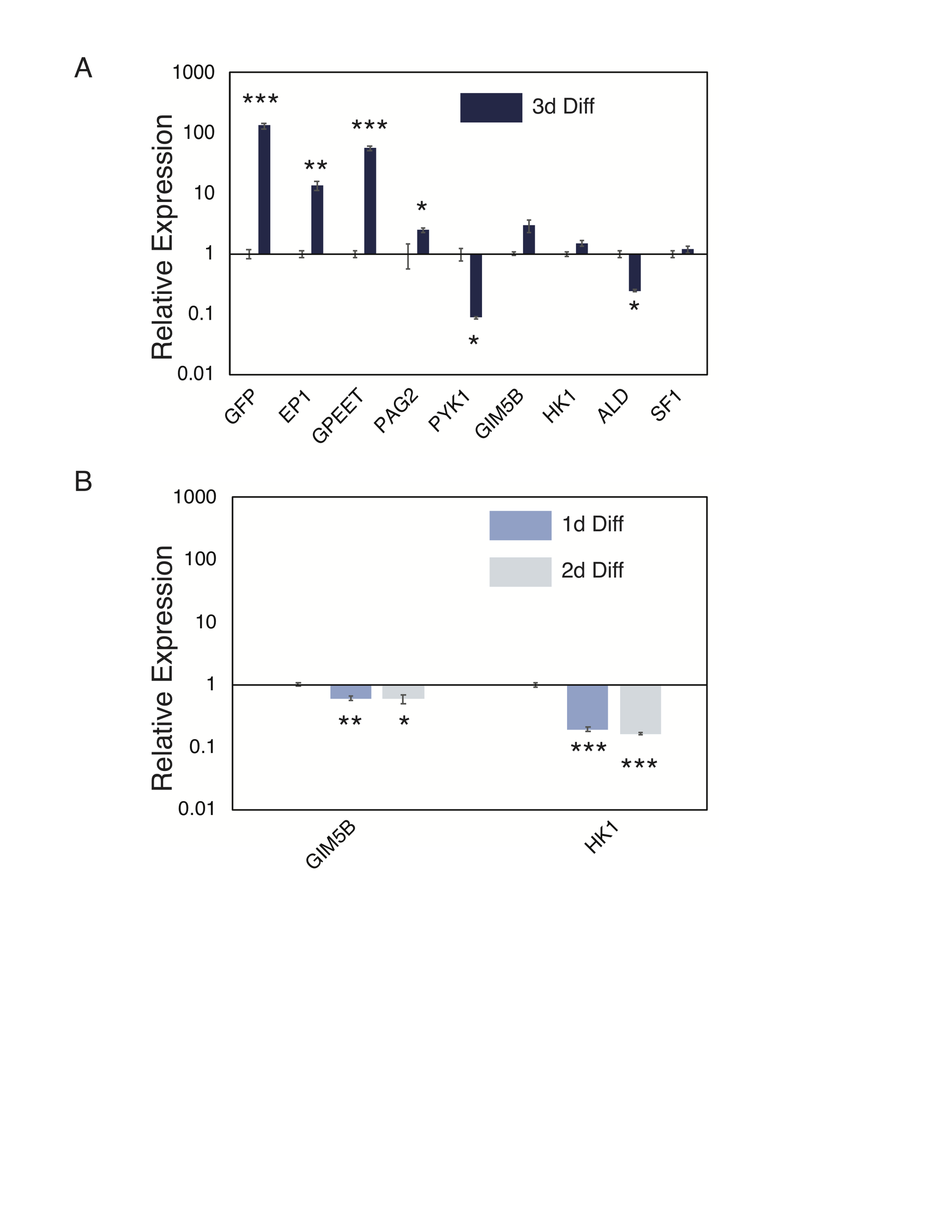

Supplement: S3 Fig — A) Gene expression for genes associated with differentiation in bloodstream parasites treated with 6mM cis-aconitate and incubated at 27°C for 3 days. B) Gene expression of HK1 and GIM5B for parasites isolated after 1 or 2 days induction of differentiation, as in A. (TIF) [file pntd.0007790.s003.tif]

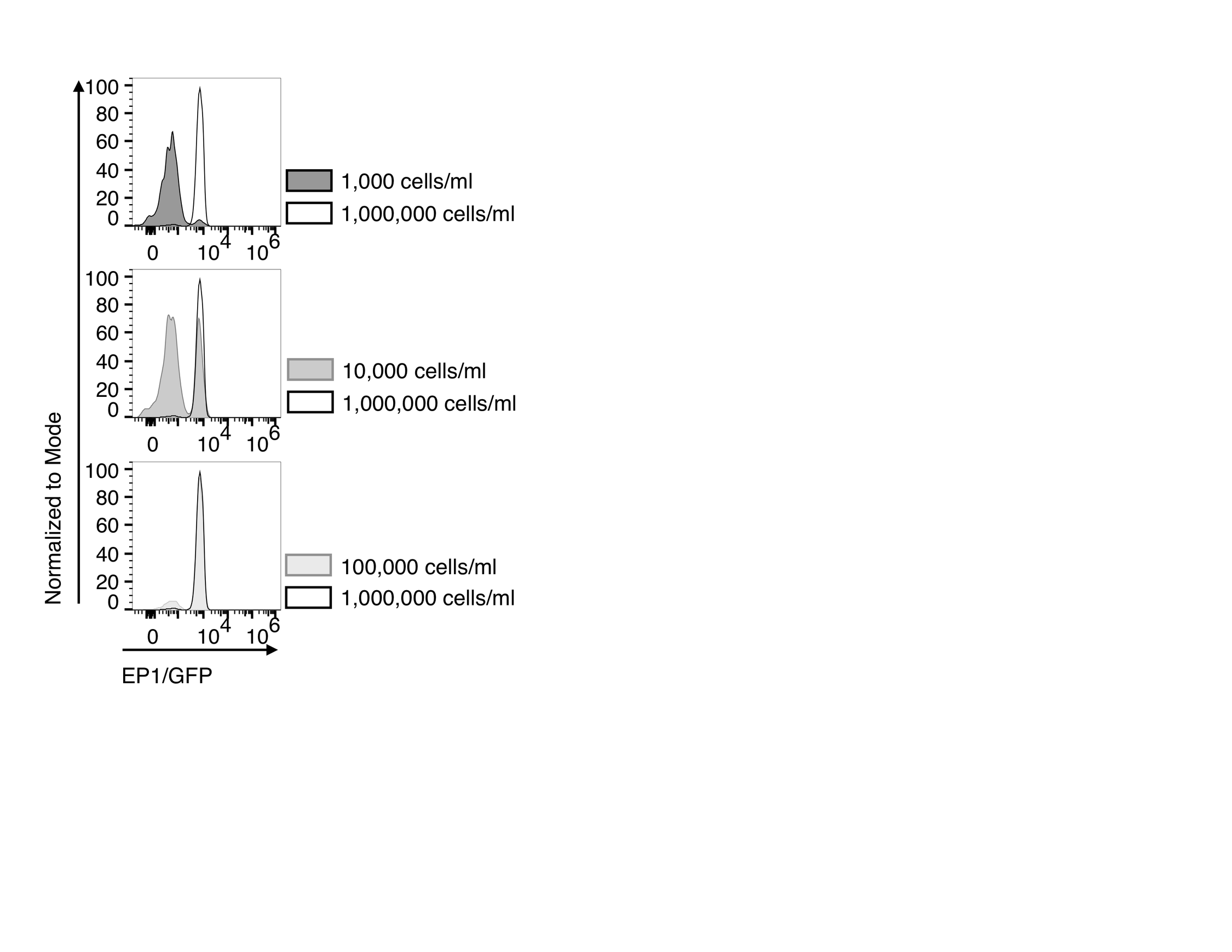

Supplement: S4 Fig — EP1/GFP expression for Antat 1.1 EP1/GFP reporter parasites seeded at 1/10 the indicated density and analyzed after 1 day of growth. (TIF) [file pntd.0007790.s004.tif]
